# Editorial: Physical therapy in the treatment of skin diseases and its mechanism

**DOI:** 10.3389/fmed.2023.1266047

**Published:** 2023-08-08

**Authors:** Michael R. Hamblin, Rui Yin, Dan Jian

**Affiliations:** ^1^Laser Research Center, Faculty of Health Sciences, University of Johannesburg, Johannesburg, South Africa; ^2^Department of Dermatology, Southwest Hospital, Army Medical University, Chongqing, China; ^3^Department of Dermatology, Central South University, Changsha, China

**Keywords:** dermatology, striae albae, infected wound healing, keloids, melasma and lasers, plantar warts

Although the pharmaceutical industry has made major leaps forward in recent years, a significant fraction of the global population does not regard these achievements as a completely unalloyed triumph. This disillusion can be explained by worries about the unwanted adverse effects of many drugs, high and rising costs making some advanced drugs unaffordable, non-availability in some developing countries, and reports of questionable marketing approaches having been carried out by “Big Pharma” within recent years (1).

All these considerations have worked together to encourage the development of non-pharmaceutical approaches by a variety of researchers, for the treatment of diseases affecting many different organ systems. These non-pharmaceutical approaches can take a variety of forms, but many can be classified under a broad heading of Physical Therapy or Energy Medicine. This involves applying external physical energy to the body that can be in the form of light, ionizing radiation, heat, cold, plasma, radiofrequency, ultrasound, electrical or magnetic fields, etc. Although physical energy can be delivered inside the body using some minimally invasive approaches, such as radiotherapy, endoscopy, implanted electrodes, or fiber optics, by far the most common approach is to deliver it from outside the body. This inevitably means that the first organ that the energy encounters as it enters the body will be the skin. Therefore, it is not surprising that the medical specialty of Dermatology has been one of the leading areas in which physical energy approaches have been explored or clinically utilized (2). The strong growth in the practice of Cosmetic Dermatology as populations have grown in affluence and the usage of social media, has encouraged this development, because there is an understandable reluctance of many patients to take drugs to improve their cosmetic appearance.

Lasers and other light sources have been employed in dermatology for many years, ever since the principle of “selective photothermolysis” was first propounded by Anderson and Parrish in 1983 (3). This principle relies on the selective absorption of pulsed light by chomophores located within the skin. The absorbed energy can be converted into highly localized heat when the laser pulse duration is shorter that the thermal relaxation time. The localized heat can selectively damage unwanted structures within the skin (blood vessels, pigmented lesions, scar tissue, tattoo pigments, etc.), without producing any generalized thermal damage. Highly sophisticated dermatology lasers and intense pulsed light sources are now available to treat a wide variety of skin conditions (4). Some of the devices employ a fractional approach, such as fractional ablative or non-ablative lasers for skin rejuvenation (5). Another approach frequently used on the face involves photobiomodulation therapy, or the use of low levels of red and/or near infrared light often delivered from safe inexpensive light emitting diodes (LEDs) (6). A different light based approach is called photodynamic therapy (PDT), and this involves combining visible light with an externally applied photosensitizer. In dermatology, this photosensitizer frequently takes the form of protoporphyrin IX naturally synthesized in the body after application of the pro-drug called 5-aminolevulinic acid (ALA), which after a few hours is activated by light from LEDs or lasers (7).

The current Research Topic of Frontiers in Medicine Dermatology has gathered together five papers (two reviews and three clinical studies) concerned with five different dermatological conditions, loosely focused around the topic of “Physical Therapy in The Treatment of Skin Diseases”. These five different clinical conditions were, infected wound healing, keloid scars, striae albae, melasma, and plantar warts.

The first review by Ning et al. discusses the ability of PDT to help with wound healing, concentrating on chronic non-healing wounds and infected wounds. PDT can act as a dual-function therapeutic approach because it can both kill the bacteria, fungi or other pathogens infecting the wound, as well as stimulating host skin cells and immune cells to enhance healing. Antimicrobial PDT mostly involves adding exogenous photosensitizers (often cationic dye molecules) into the infected wound and then irradiating the wound with light. Host stimulation of healing is often based on ALA-PDT. The review mainly summarizes clinical studies, but some animal models of infected wounds are also included.

The second review by Wang et al. is concerned with the use of ionizing radiation for the treatment of keloids. Keloids are benign skin tumors composed of fibroblasts and collagen, and are often associated with an abnormal healing response of skin wounds in susceptible individuals. Ionizing radiation can involve the external application of X-rays, electron beam therapy, or heavy ion beam therapy (carbon ions). Brachytherapy involves interstitial implantation of radionuclides into the tissue. Both approaches aim to kill the abnormal fibroblasts responsible for synthesizing the excess collagen within the keloid. The main problem encountered is the recurrence of the lesion, which could be as high as 40% depending on the specific trial.

The first clinical study was by Luo et al., who compared the use of two fractional lasers (1,500 nm Er:Glass and 10,600 nm CO_2_) for the treatment of striae albae. Striae albae are disfiguring stretch marks on the abdomen caused by pregnancy, obesity, or rapid weight gain/loss. Twenty-seven subjects were recruited. Each side of the abdomen was treated with a different laser, three times at 2-month intervals, with 3 months follow-up. The Er:Glass laser gave significantly better results compared to the CO_2_ laser, with respect to overall appearance, patient acceptability and less hyperpigmentation as a side effect.

The next clinical study was by Liang et al., who also compared the effectiveness of two different non-fractional picosecond pulsed lasers (1,064 nm Nd:YAG and 755 nm alexandrite), this time for the treatment of melasma. Melasma is the disfiguring occurrence of hyperpigmented patches, which are often found in sun-exposed skin, or arise in women due to pregnancy or the action of birth control hormones. These workers recruited 60 melasma patients who were split into three groups. The two laser groups were treated with three laser sessions at 4-week intervals, while the third group was treated with 2% hydroquinone cream (standard treatment) applied twice daily for 12 weeks. The melasma area and severity index (MASI) scores, were evaluated at weeks 0, 4, 8, 12, 16, 20, and 24. The ps Nd:YAG laser group showed significantly better improvements in the MASI score compared with the other two groups, as well as higher patient satisfaction.

The final clinical study was by Chen et al. who reported a pilot clinical trial of local hyperthermia for treating plantar warts in 38 patients. They employed a light source delivering a focused infrared beam designed to raise the skin temperature of the lesion to 45°C for 30 min. Patients received treatment over a 5-week schedule, on days 1, 2, 3, 14, 15, 22, 29, and 36. Of the 38 patients, complete resolution of the warts occurred in 13 (34.2%), 8 (21.1%) achieved partial remission, while 17 (44.7%) showed a poor response to the treatment. In the 13 patients who achieved a complete response there was no recurrence over a 3 month follow up.

## Author contributions

MH: Writing—original draft. RY: Writing—review and editing. DJ: Writing—review and editing.
